# An E2F1/DDX11/EZH2 Positive Feedback Loop Promotes Cell Proliferation in Hepatocellular Carcinoma

**DOI:** 10.3389/fonc.2020.593293

**Published:** 2021-02-05

**Authors:** Shu-Guang Su, Qiu-Li Li, Mei-Fang Zhang, Peng-Wei Zhang, Huimin Shen, Chris Zhiyi Zhang

**Affiliations:** ^1^ Department of Pathology, The Affiliated Hexian Memorial Hospital of Southern Medical University, Guangzhou, China; ^2^ Department of Head and Neck Surgery, Sun Yat-sen University Cancer Center, Guangzhou, China; ^3^ Department of Pathology, Sun Yat-sen University Cancer Center, Guangzhou, China; ^4^ MOE Key Laboratory of Tumor Molecular Biology and Key Laboratory of Functional Protein Research of Guangdong Higher Education Institutes, Institute of Life and Health Engineering, College of Life Science and Technology, Jinan University, Guangzhou, China; ^5^ Department of Obstetrics and Gynecology, The First Affiliated Hospital of Sun Yat-sen University, Guangzhou, China

**Keywords:** DDX11, E2F1, EZH2, p21, hepatocellular carcinoma

## Abstract

Hepatocellular carcinoma (HCC) accounts for one of the leading causes of cancer-related death, and is attributed to the dysregulation of genes involved in genome stability. DDX11, a DNA helicase, has been implicated in rare genetic disease and human cancers. Yet, its clinical value, biological function, and the underlying mechanism in HCC progression are not fully understood. Here, we show that DDX11 is upregulated in HCC and exhibits oncogenic activity *via* EZH2/p21 signaling. High expression of DDX11 is significantly correlated with poor outcomes of HCC patients in two independent cohorts. DDX11 overexpression increases HCC cell viabilities and colony formation, whereas DDX11 knockdown arrests cells at G1 phase without alteration of p53 expression. Ectopic expression of DDX11 reduces, while depletion of DDX11 induces the expression of p21. Treatment of p21 siRNA markedly attenuates the cell growth suppression caused by DDX11 silence. Further studies reveal that DDX11 interacts with EZH2 in HCC cells to protect it from ubiquitination-mediated protein degradation, consequently resulting in the downregulation of p21. In addition, E2F1 is identified as one of the upstream regulators of DDX11, and forms a positive feedback loop with EZH2 to upregulate DDX11 and facilitate cell proliferation. Collectively, our data suggest DDX11 as a promising prognostic factor and an oncogene in HCC *via* a E2F1/DDX11/EZH2 positive feedback loop.

## Introduction

Hepatocellular carcinoma (HCC), accounting for the majority of primary liver cancer, is the second leading cause for cancer-related death in many parts of the world (1). The global burden of HCC has been increasing for decades and the morbidity may soon surpass one million cases per year (2). Over half of the patients with HCC were diagnosed at advanced-stage disease, and therefore must receive systemic treatments, to which rare significant progress has been made (3, 4). Therefore, the improvement of the prognosis of HCC patients is modest and limited. Genomic and transcriptomic studies provide novel insights into the biological mechanism of the development and progression of HCC (3, 4). Further studies are required to identify potential prognostic and therapeutic biomarkers.

Genes involved in DNA replication and proper sister chromatic cohesion are essential for the cell division. Dysregulation of such genes contribute a great deal to the initiation and progression of malignant diseases. DDX11, also known as Chl1 or ChlR1, belongs to super family 2 (SR2) ATP-dependent DEAD (Asp–Glu–Ala–Asp)/DEAH (Asp– Glu–Ala–His) helicase (5, 6). DDX11 shares similar sequence with DNA helicases FANCJ and XPD and plays important role in genome stability maintenance (7). Mutation of DDX11 is considered as the cause of rare genetic disease Warsaw breakage syndrome (WABS) (8). DDX11 deficiency is also response for aneuploidy, cohesion defects, and cell apoptosis (8). Due to the essential regulation in maintenance of sister chromatin cohesion, DDX11 exerts biological functions in cell cycle modulation (9, 10). Interaction with other proteins, such as RAD17 (11) and Timeless (12, 13), was the important manner for DDX11 to exhibit biological activities. Upregulation of DDX11 was documented in lung adenocarcinoma (14), osteosarcoma (15), advanced melanoma (16), and HCC (17), which indicate that DDX11 may exert pro-tumor activity. Furthermore, DDX11 was identified as one of the determinants for the sensitivity of cancer cells to PARP inhibitors (18). However, the detailed mechanism through which DDX11 participates in the tumorigenesis and cancer progression remains largely unknown.

Using tissue microarray (TMA)-based pathological detection and *in vitro* experiments, we intended to determine the expression and clinical significance of DDX11 in HCC, and investigate the biological role and underlying mechanism of DDX11 in HCC progression. Our data suggest DDX11 as a potential unfavorable biomarker for patients’ prognosis, and an oncogene to promote cell proliferation *via* EZH2/p21 signaling.

## Materials and Methods

### Patients and Specimens

All patients in this study provided written informed consents and did not received chemotherapy or radiotherapy before surgical resection. Our study was approved by the Institute Research Ethics Committee of The Affiliated Hexian Memorial Hospital of Southern Medical University. Clinical samples were collected in Sun Yat-sen university and The Affiliated Hexian Memorial Hospital of Southern Medical University, including 24 pairs of fresh HCC specimens and 386 archived paraffin-embedded HCC tissues. The increased expression of DDX11 in HCC was validated by studies of Gene Expression Omnibus (GEO), Oncomine, and The Cancer Genome Atlas (TCGA).

### Gene Set Enrichment Analysis

Gene set enrichment analysis (GSEA) was conducted using the java (GSEA 4.1.0)–based graphical user interface using a pre-ranked list with the classic enrichment statistic. GSEA involves determining whether a predefined set of genes is significantly different between the two groups: high and low DDX11. The entire list of genes is ranked according to expression difference of DDX11 in HCC samples form TCGA database. An enrichment score for each gene set is then calculated. Cumulative distribution function was constructed by performing 1,000 random gene set membership assignments. The following gene sets databases were applied: h.all.v7.2.symbols.gmt (Hallmark), c2.cp.biocarta.v7.2.symbols.gmt (BIOCARTA), c2.cp.kegg.v7.2.symbols.gmt (KEGG) and c6.all.v7.2.symbols.gmt (Oncogenic)

### Cell Culture and Transfection

HepG2 and PLC8024 cells were purchased from the Cell Resource Center, Chinese Academy of Science Committee (Shanghai, China), and cultured by Dulbecco’s modified Eagle’s medium (DMEM) (Gibco, Gaithersburg, MD, USA) supplemented with 10% heat-inactivated fetal bovine serum (FBS, Hyclone, Logan, UT). Stable cell lines were constructed by transfecting cells with pcDNA (3.1) overexpression vector encoding full-length DDX11 cDNA or DDX11 shRNA purchased from Santa-cruz company (sc-77104-SH), and then selected by G418 for two weeks. siRNAs targeting p21 (#6456, Cell Signaling Technology), E2F1 (sc-29297, Santa-cruz Biotechnology), E2F2 (sc-29298, Santa-cruz Biotechnology), E2F3 (sc-37817, Santa-cruz Biotechnology), and EZH2 (#6509, Cell Signaling Technology) were also transiently introduced into HCC cells.

### Quantitative Real-Time Polymerase Chain Reaction (qRT-PCR)

The mRNA expression level of DDX11, EZH2, E2F1, E2F2, and E2F3 was determined by qRT-PCR, using a SYBR Green real-time assay. Total RNAs were extracted from cells using the Trizol reagent (Invitrogen, CA, USA) according to the manufacturer’s instruction. One microgram of RNA sample was reverse transcribed using the Superscript III enzyme (Invitrogen, CA, USA) to obtain single-stranded cDNA. Real-time PCR was then performed on cDNA in an iQ Sybr Green Supermix (Bio-Rad) with gene-specific primers. The following primers were used: DDX11, forward: 5’- ATTTGTGGGGCCCTCTCTTG-3’ and reverse: 5’-AAACTGAGTAACCCAGGCCG-3’; EZH2, forward: 5’- GCCAAATATTGAACCTCCTG-3’ and reverse: 5’-AAACATGGTTAGAGGAGCCG-3’; E2F1, forward: 5’- GCCACCATGGCCTTGGCCGG-3’ and reverse: 5’-AAGCCCTGTCAGAAATCCAG-3’; E2F2, forward: 5’- GAGCTCACTCAGACCCCAAG-3’ and reverse: 5’-AACAGGCTGAAGCCAAAAGA-3’; E2F3, forward: 5’- TGACCCAATGGTAGGCACAT-3’ and reverse: 5’-CATCTAGGACCACACCGACA-3’; β-actin, forward: 5′-TGGCACCCAGCACAATGAA-3′ and reverse: 5′-CTAAGTCATAGTCCGCCTAGAAGCA-3′.

### Western Blot

Western blot experiments were performed according to the instruction of our previous studies (19). The information of antibodies used in this study was given as followings: DDX11 (ab230017, Abcam), E2F1 (#3742, cell signaling technology), E2F2 (#ab138515, Abcam), E2F3 (#ab50917, Abcam), EZH2 (#5246, cell signaling technology), p21 (#2947, cell signaling technology), p53 (#2524, cell signaling technology), phosphorylated Rb (#8147, cell signaling technology), cyclin E (#4136, cell signaling technology), cyclin D1 (#55506, cell signaling technology), MDM2 (#86934, cell signaling technology), β-actin (#3700, cell signaling technology).

### Immunohistochemistry (IHC)

IHC was used to examine the expression of DDX11 in HCC tissues. Paraffin embedded sections were dewaxed in xylene (3 × 5 min) and dehydrated in ethanol series (3 min in 100% ethanol, 1 min in each of 95% and 70% ethanol). Sections were washed in PBS and endogenous peroxidases were blocked with 3% H2O2 for 10 min. The tissue sections were subjected to antigen retrieval by pressured cooking in 10 mM citrate buffer for 3 min, and then incubated with serum blocking solution for 20 min to block nonspecific binding, followed by incubation with primary antibodies for 2 h at room temperature. After rinsing in PBS for 10 min, the sections were incubated with the biotinylated secondary antibody for 1 h and further incubation with the Streptavidin Biotin complex. Reactivity was developed in chromogen DAB (3,3-diaminobenzidine) solution. The signal was enhanced by applying the solution of CuSO4 and NaCl for 5 min. Finally, the sections were counterstained with Mayer’s hematoxylin, dehydrated, and mounted. All sections were observed under light microscopy and the staining intensities were assessed by two independent pathologists (Yang YF and Cao Y). Nucleus staining was graded for intensity (0-negative, 1-weak, 2-moderate, and 3-strong) and percentage of positive cells [0, 1 (1–24%), 2 (25–49%), 3 (50–74%), and 4 (75–100%)] with discrepancies resolved by consensus. The H-scores for tumors with multiple cores were averaged. The median IHC score was used to define high DDX11 expression and low DDX11 expression groups.

### Colony Formation

Stable cells with DDX11 overexpression or knockdown were culture in 6-well plates at a density of 1.0 × 10^3^ per well by medium plus G418 for 10 days. Colonies were fixed with methanol, stained with 0.1% crystal violet, pictured, and counted under a microscope.

### Statistics

Difference of TRAIP expression in HCC and nontumorous tissues was revealed by Student’s t-test. Data are mean and SEM from three independent *in vitro* experiments. Significance between groups was calculated by the Student’s t-test. Survival analyses were conducted by Kaplan-Meier analyses (log-rank test). Differences were considered significant for *P*-values less than 0.05.

## Results

### DDX11 Is Upregulated in HCC and Correlated With Poor Prognosis

**Figure 1:**
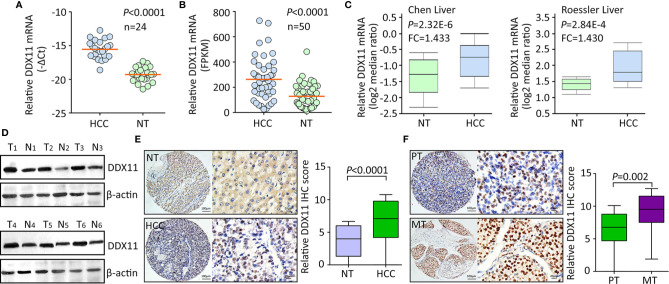
DDX11 expression is increased in HCC tissues. **(A)** The mRNA expression of DDX11 was determined in 24 pairs of fresh HCC and nontumorous (NT) samples, using qRT-PCR in SYSUCC cohort (Matched pair t test). **(B)** The increased expression of DDX11 mRNA was validated in 50 paired HCC tissues in TCGA cohort (Matched pair t test). **(C)** GEO studies indicate the level of DDX11 mRNA in HCC cases was higher than that in nontumorous (NT). **(D)** Proteins extracted in clinical samples were subjected to western blot to examine the DDX11 protein expression. β-actin was used as a loading control. T, tumor samples; N, nontumorous samples. **(E)** A large cohort containing 328 HCC cases was recruited. The protein level of DDX11 was measured by IHC in paraffin-embedded tissues. Representative images and relative IHC scores were presented in the left and right panels, respectively. **(F)** Another cohort consisting of 47 paired primary HCC and corresponding portal vein tumor thrombus tissues was collected to detect the expression of DDX11 by IHC.

Using TCGA data, we firstly assessed the expression of DDX11 in TCGA gastrointestinal cancers, including liver hepatocellular carcinoma (LIHC), colon adenocarcinoma (COAD), esophageal carcinoma (ESCA), pancreatic adenocarcinoma (PAAD), rectum adenocarcinoma (READ), and stomach adenocarcinoma (STAD). Results showed the biggest difference of DDX11 expression occurred in HCC (**Supplementary Figure 1**). To further determine the expression of DDX11 in HCC, we collected 24 pairs of fresh HCC specimens. qRT-PCR results showed that DDX11 mRNA was significantly increased in HCC tissues, compared with the corresponding adjacent nontumorous tissues (**Figure 1A**). Analyses based on TCGA and Oncomine data validated the upregulation of DDX11 mRNA in HCC tissues. Studies of TCGA, Chen liver, Roessler liver reported the increase of DDX11 in HCC (**Figures 1B, C**). Consistently, DDX11 protein expression in HCC was much higher than that in nontumorous tissues. In 24 pairs of fresh clinical specimens, DDX11 expression was noticeably increased (**Figure 1D**). This was confirmed by IHC detection of DDX11 protein expression in 328 paraffin-embedded tissues. DDX11 mainly localized in the nuclear of cancer cells (**Figure 1E**). About 69.5% (228/328) of patients expressed more DDX11 in HCC tissues. Strikingly, strong expression of DDX11 was frequently depicted in metastatic tissues, compared to the primary tissues (**Figure 1F**). These data suggest that DDX11 is upregulated in HCC.

**Figure 2:**
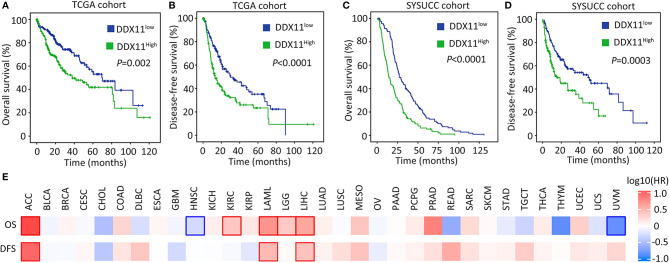
High expression of DDX11 is correlated with poor prognosis in HCC. **(A, B)** Patients with HCC in TCGA cohort were divided into DDX11^high^ and DDX11^low^, according to the median expression of DDX11 mRNA. Kaplan-Meier survival analyses (log-rank test) were conducted to indicate the clinical value of DDX11 mRNA in overall **(A)** and disease-free **(B)** survivals. **(C, D)** The implication of DDX11 protein expression was determined in patients with HCC in SYSUCC cohort. The median IHC score of DDX11 in HCC tissues was used as the cut-off value to separate cases into groups of DDX11^high^ and DDX11^low^. **(E)** The impact of DDX11 mRNA on overall survival (OS) and disease-free survival (DFS) was tested in TCGA cancers (Mantel–Cox test). Significant *p* values were indicated by red and blue frames.

We next determined the clinical significance of DDX11 overexpression in HCC. Patients in TCGA cohort were divided into DDX11^high^ and DDX11^low^ groups, according to the median value of mRNA expression. Kaplan-Meier analyses indicated that high expression of DDX11 mRNA was correlated with poor overall and disease-free survivals (**Figures 2A, B**). The prognostic value of DDX11 was next validated in SYSUCC cohort. The median IHC score of DDX11 in HCC tissues was used to separate the patients. Patients with high DDX11 expression were likely accompanied with shorter overall survival and experienced tumor relapse in shorter time (**Figures 2C, D**). In addition, our data further showed that DDX11 mRNA expression was able to distinguish the clinical outcomes in several TCGA cancers, such as adrenocortical carcinoma (ACC) and acute myeloid leukemia (LAML) (**Figure 2E**). These data suggest DDX11 as a potent prognostic factor in human cancers.

### DDX11 Promotes Cell Proliferation in HCC

**Figure 3:**
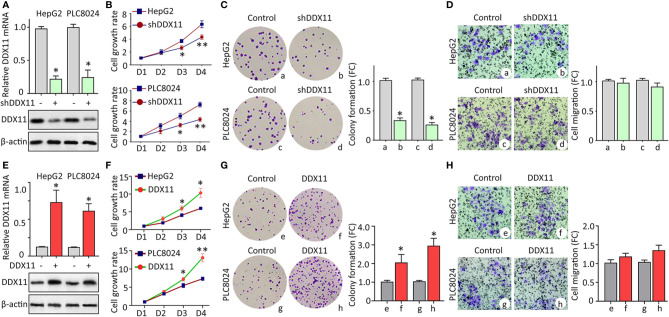
DDX11 promotes cell proliferation in HCC. **(A)** Cells were transfected with DDX11 shRNA and selected by G418 to construct stable cell lines with DDX11 knockdown. qRT-PCR and western blot were used to confirmed the downregulation of DDX11 mRNA and protein in HepG2 and PLC8024 cells. *P < 0.05. **(B)** The effect of DDX11 silence on HCC cell growth were determined by MTT assays. Stable cells were placed into 96-well plates to continue to grow for 4 days. Cell growth rates were calculated by the absorbance at OD490 nm. *P < 0.05, **P < 0.01.* C*. Colony formation was performed to validate the role of DDX11 on cell proliferation. Stable cells were cultured in 60 mm plates with G418-contained DMEM for 10 days. Colonies were pictured and counted under a microscope (left panel). The fold change (FC) of colony formation was indicated by histogram (right panel). *P < 0.05. **(D)** Transwell assays were performed to assess whether DDX11 affects cell migration. Cells transferred to the bottom side of the Transwell chamber were stained by 0.1% crystal violet and counted. The fold change (FC) of cell migration were shown. *P < 0.05. **(E–H)** Stable cells with DDX11 overexpression were constructed **(E)** and used in the determination of DDX11-mediated cell growth by MTT **(F)**, colony formation **(G)** and Transwell **(H)** assays. *P < 0.05, **P < 0.01.

Clinicopathological data suggest DDX11 might be involved in the progression of HCC. We next intended to disclose the biological function in HCC cells. According to the expression of DDX11 in HCC cell lines (data not shown), HepG2 and PLC8024 were chosen for the further investigation. Specific shRNA targeting DDX11 was transfected into HCC cells to construct stable cell lines with DDX11 knockdown. qRT-PCR and western blot were performed to confirm the downregulation of DDX11 (**Figure 3A**). MTT assays showed that silence of DDX11 led to the inhibition of cell growth (**Figure 3B**). A markedly decrease of colony formation was observed in cells with DDX11 depletion (**Figure 3C**). Since DDX11 was overexpressed in metastatic tissues, compared with the primary tissues (**Figure 1F**). Its effect on cell migration was determined. Transwell assays showed no significant difference of the migrated cells, with or without DDX11 knockdown. These data indicated that DDX11 may functions as an oncogene to promote cell proliferation. To prove this assumption, we overexpressed DDX11 in HepG2 and PLC8024 cells (**Figure 3E**). MTT and colony formation demonstrated that DDX11 overexpression dramatically enhanced the proliferative ability of HCC cells (**Figures 3F, G**), but did not affect the metastatic potent (**Figure 3H**).

### DDX11 Depletion Induces Cell Cycle Arrest in HCC Cells *via* p21

**Figure 4:**
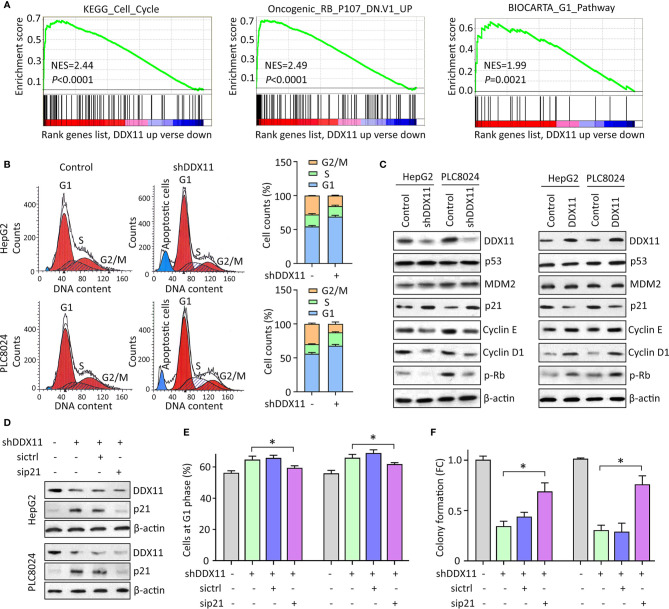
DDX11 depletion induces G1 phase arrest in HCC cells. **(A)** GSEA based on TCGA data indicated that in cases with high expression of DDX11, pathways involved in cell cycle regulation were activated or suppressed. **(B)** Cells with DDX11 knockdown by shRNA were stained with PI and subjected to cytometry analyses to indicate the alteration of cell cycle. The percentages of cells at each cell cycle or under apoptosis were shown. **(C)** The expressions of factors contributing to cell cycle regulation, such as p53, MDM2, p21, cyclin E, cyclin D1, and phosphorylated Rb were determined by western blot, in cells with DDX11 silence or overexpression. **(D)** Stable HepG2 and PLC8024 cells were transfected with p21 siRNA for 36 h. The expression of p21 and DDX11 was determined. **(E, F)** The effect of p21 on DDX11-mediated G1 phase arrest and cell growth suppression was examined by rescue experiments. Stable cells with DDX11 silence and/or p21 knockdown were subjected to cytometry analyses **(E)** and colony formation **(F)**. *P < 0.05.

We were next interested in the reason of DDX11-mediated cell proliferation. GSEA data indicated that signaling pathways involved in the regulation of cell cycle were significantly modulated in patients with high expression of DDX11 (**Figure 4A**). As a result, we next checked the alteration of cell cycle in HCC cells with DDX11 knockdown. Data of flow cytometry presented a marked increase of cells at G1 phase, upon to the treatment of DDX11 shRNA (**Figure 4B**). Upregulation of p21 and downregulation of phosphorylated Rb, cyclin E and cyclin D1 were detected in cells with DDX11 knockdown. In contrast, p21 was reduced in cells with DDX11 overexpression (**Figure 4C**). However, the expression of p53 and MDM2 remained unchanged (**Figure 4C**), which may suggest a p53-independent manner of DDX11-mediated p21 alteration.

We next determined the role of p21 in DDX11-mediated malignant phenotype. p21 was knocked down in cells with DDX11 depletion (**Figure 4D**). G1 phase arrest induced by DDX11 shRNA was partly released by the p21 knockdown in both HepG2 and PLC8024 cells (**Figure 4E**). Consequently, the suppression of cell proliferation caused by DDX11 shRNA was attenuated by the downregulation of p21 (**Figure 4F**). Collectively, these data suggest that DDX11 promotes HCC progression *via* p21 suppression.

### DDX11 Downregulates p21 *via* Enhancing the Protein Stability of EZH2

**Figure 5:**
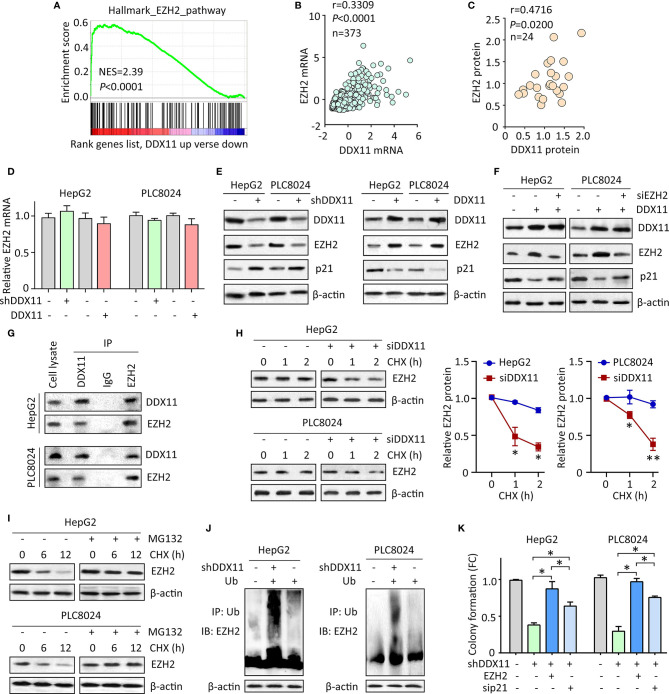
DDX11 suppresses p21 *via* enhance the protein stability of EZH2 in HCC cells. **(A)** GSEA indicated that EZH2 signaling was activated in patients with high expression of DDX11. **(B)** In TCGA cases, a positive correlation between DDX11 mRNA and EZH2 mRNA expression was found. **(C)** The protein expression of DDX11 was associated with EZH2 protein expression in 24 fresh HCC specimens in SYSUCC cohort. **(D)** HepG2 and PLC8024 cells were transfected with DDX11 shRNA or overexpression vectors. The mRNA expression of EZH2 was examined by qRT-PCR. **(E)** The expression of DDX11, EZH2 and p21 in stable cells with DDX11 knockdown or overexpression was examined by western blot. **(F)** DDX11-expressing cells were transfected with EZH2 siRNA for 36 h. The effect of EZH2 on DDX11-mediated p21 suppression was tested. **(G)** The protein binding of EZH2 and DDX11 was confirmed by co-IP experiments. **(H)** The EZH2 protein stability was measured by CHX treatment in cells with DDX11 knockdown. *P < 0.05, **P < 0.01. **(I)** Cells were cultured with 20 mg/L CHX for indicated time, with or without pretreatment of MG132 (20 μM). The protein degradation of EZH2 was determined by western blot. **(J)** HCC cells with or without DDX11 knockdown were transfected with Ub for 48 h. The ubiquitination of EZH2 protein was examined by co-IP mediated by Ub antibody. **(K)** The role of EZH2/p21 axis was assessed by rescue experiment, using colony formation. *P < 0.05.

We then planned to disclose the mechanism of DDX11-mediated p21 suppression. Literatures reported that p21 could be epigenetically regulated by EZH2 *via* its histone methyltransferase activity in a p53-independent fashion (20). GSEA showed the activation of EZH1 signaling in HCC cases with DDX11 upregulation (**Figure 5A**). In TCGA HCC tissues, a positive correlation of DDX11 mRNA and EZH2 mRNA was found (**Figure 5B**). Further, we also observed that DDX11 protein expression was closely associated with EZH2 protein expression in 24 HCC specimens (**Figure 5C**). We therefore postulated that DDX11 may modulate the expression of p21 in HCC cells *via* EZH2. No significant change of EZH2 mRNA expression was detected in stable cells with DDX11 knockdown or overexpression (**Figure 5D**). Strikingly, EZH2 protein was downregulated in cells with DDX11 depletion, and upregulated in cells with DDX11 overexpression (**Figure 5E**), suggesting a post-transcriptional regulation of EZH2 by DDX11. To reveal the role of EZH2 in DDX11-mediated p21 suppression, we knocked down EZH2 in DDX11-expressing cells. The expression of p21 was comparable to the control cells, after the knockdown of EZH2 (**Figure 5F**). These findings indicated that DDX11 inhibited p21 expression *via* EZH2.

Previous studies reported DDX11 exerted biological function *via* interacting with other proteins (11, 13). We next examined whether DDX11 bound to EZH2 in HCC cells. coIP experiments were performed to showed that DDX11 and EZH2 were detectable in precipitant mediated by specific antibody for DDX11 or EZH2 in both HCC cells (**Figure 5G**). We next determined whether this interaction resulted in the upregulation of EZH2. After treatment of Cycloheximide (CHX), the EZH2 protein was rapidly degradation in HCC cells with DDX11 knockdown (**Figure 5H**). Upon the treatment of 20 μM of MG132, an inhibitor of protein ubiquitination, the degradation of EZH2 was inhibited (**Figure 5I**). Thus, we next checked whether the binding of DDX11 and EZH2 may affect the ubiquitination of EZH2 in HCC cells. Results demonstrated that the ubiquitination of EZH2 protein was obviously enhanced by the depletion of DDX11 (**Figure 5J**). Functionally, ectopic expression of EZH2 or p21 knockdown could reverse the suppression of colony formation by shDDX11 (**Figure 5K**). Collectively, the above data suggest that EZH2/p21 axis was essential for the biological function of DDX11 in HCC cells.

### E2F1/DDX11/EZH2 Forms a Positive Feedback Loop in HCC Cells

**Figure 6:**
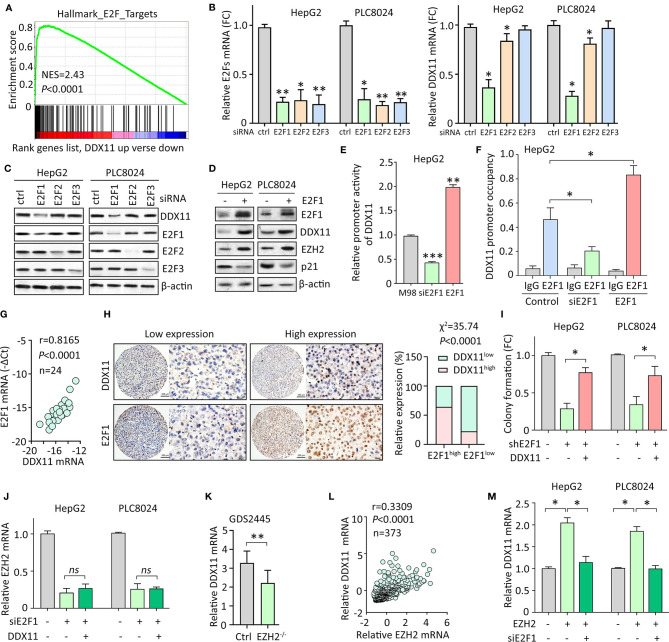
E2F1/DDX11/EZH2 forms a positive feedback loop in HCC cells. **(A)** GSEA indicated that DDX11 may be a downstream target of E2F transcription factors. **(B, C)** HepG2 and PLC8024 cells were transfected with siRNAs for E2F family members, including E2F1, E2F2, and E2F3. The expression of E2Fs and DDX11 mRNA was determined by qRT-PCR **(B)** and western blot **(C)**. **(D)** E2F1 was overexpressed in HCC cells. Expression of E2F1, DDX11, EZH2, and p21 was examined. **(E)** Dual luciferase reporter assays were performed in HepG2 cells with E2F1 overexpression or knockdown to indicate the effect of E2F1 on the activity of DDX11 promoter. **P < 0.01, ***P < 0.001. **(F)** ChIP assays were used to detect the enrichment of E2F1 on DDX11 promoter. *P < 0.05. **(G)** Correlation between DDX11 mRNA and E2F1 was determined in 24 HCC tissues (Pearson correlation analysis). **(H)** The positive correlation of E2F1 and DDX11 protein expression was confirmed in 303 paraffin-embedded HCC tissues. Patients with high expression of E2F1 were accompanied with more DDX11 expression. **(I)** Cells with E2F1 silence were transfected with DDX11 overexpression vector. Colony formation was performed to examine the role of DDX11 in shE1F1-mediated cell growth suppression. *P < 0.05. **(J)** Cells were transfected with E2F1 siRNA and DDX11 overexpression vector for 36 h. The mRNA expression of EZH2 was examined. ns, not significant. **(K)** According to the published data (GDS2445), DDX11 mRNA was downregulated in EZH2^-/-^ cells. **(L)** The association of EZH2 and DDX11 was determined in TCGA cases. **(M)** Cells were overexpressed with EZH2 and/or knockdown of E2F1. The mRNA expression of DDX11 was examined. *P < 0.05.

We next intended to identify the upstream regulator of DDX11 in HCC. GSEA suggested DDX11 may be one of the downstream effectors of E2Fs transcriptional factors (**Figure 6A**). According to TCGA data, positive correlation was found between DDX11 and E2F1, E2F2 and E2F3 (**Supplementary Figure 2**). As a result, we knocked down the expression of E2F1, E2F2, and E2F3, using specific siRNAs. A markedly decrease of DDX11 was detected in HepG2 and PLC8024 cells with E2F1 knockdown (**Figure 6B**). Western blot showed that DDX11 expression was downregulated by siE2F1 (**Figure 6C**). Furthermore, ectopic expression of E2F1 induced the expression of DDX11, consequently inducing EZH2 and reducing p21 (**Figure 6D**). We next determined whether E2F1 directly upregulated DDX11 in HCC cells. Luciferase reporter assay showed that the activity of DDX11 promoter was affected by the alteration of E2F1 expression (**Figure 6E**). ChIP assays demonstrated that E2F1 directly bound on the promoter of DDX11 (**Figure 6F**). These data indicated E2F1 was able to transcriptionally upregulate DDX11 in HCC cells. In clinical samples, DDX11 mRNA was associated with E2F1 mRNA (**Figure 6G**). HCC patients with high expression of E2F1 were usually accompanied with more DDX11 expression (**Figure 6H**). These data implied the transcriptional regulation of DDX11 by E2F1. Rescue experiments showed that the shE2F1-caused inhibition of colony formation was partly attenuated by DDX11 (**Figure 6I**), indicating that DDX11 functioned as a downstream effector of E2F1 in HCC progression.

Current studies showed E2F1 is also the transcriptional factor for EZH2. We next intended to check if DDX11 was involved in the regulation of EZH2 by E2F1. In HCC cells, knockdown of E2F1 significantly decreased the mRNA expression of EZH2. However, DDX11 overexpression was not able to rescue the EZH2 transcript (**Figure 6J**). This manifests DDX11 was not required for the E2F1-mediated EZH2 regulation. Several studies demonstrated that EZH2 could interacted with E2F1 to enhance its transcriptional activity. A GEO study (GDS2445) showed that DDX11 mRNA was upregulated in cells with EZH2 knockout (**Figure 6K**). In addition, EZH2 mRNA was positively correlated with DDX11 mRNA in TCGA patients (**Figure 6L**). We thus speculated that EZH2 may cooperate with E2F1 to induce DDX11 transcription. As expected, DDX11 mRNA was upregulated by EZH2 overexpression, which was abolished by the knockdown of E2F1 (**Figure 6M**). Taken together, our data suggest a positive feedback loop of E2F1/DDX11/EZH2 axis promotes cell proliferation in HCC.

## Discussion

DNA helicases play essential roles in the maintenance of genomic stability (21). Deficiency or loss of expression of DEAD/H-box helicase family has been demonstrated to be the cause of genetic diseases, such as Xeroderma pigmentosum, Cockayne’s syndrome, Trichothiodystrophy, or COFS syndrome (21). Mutations of DDX11 are linked to Warsaw Breakage syndrome, but not frequent in human cancers. Here, we showed that DDX11 functioned as an oncogene to promote cell proliferation through activating EZH2/p21 signaling in HCC cells. DDX11 expression was upregulated by the positive feedback loop of EZH2 and E2F1. The E2F1/DDX11/EZH2 represents a promising therapeutic target in the clinical management of HCC that is one of the leading causes of cancer-related death globally.

The prognostic value and biological function of DDX family proteins have been well documented in human cancers. For example, DDX5 interacted with autophagic receptor p62 to trigger cell autophagy and suppressed liver tumorigenesis (22). HCC patients with low DDX5 expression showed poor prognosis. DDX3 was frequently downregulated in hepatitis B virus (HBV)-positive HCC samples, served as a favorable prognostic factor, and modulated the cell cycle progression and cell apoptosis *via* p21 (23). DDX6 was overexpressed in hepatitis C virus (HCV)-HCC tissues and promoted tumor growth *via* controlling the replication of HCV in cancer cells (24). Our study provided compelling evidence that DDX11 expression was markedly increased in HCC tissues, compared with the corresponding nontumorous tissues. Furthermore, DDX11 expression in HCC samples was not associated with well-known HCC risk factors, such as alcohol consumption and hepatitis infection (**Supplementary Figure 3A**). In cirrhotic tissues adjacent to HCC, DDX11 was negatively or weakly stained by IHC (**Supplementary Figure 3B**). High DDX11 expression was significantly correlated with poor outcomes, including overall and disease-free survivals. Functionally, DDX11 promoted cell proliferation by inducing the expression of EZH2, a famous oncogene (20, 25), to subsequently inhibit the expression of p21, a well-known tumor suppressor. In line with our data, a resent paper reported that DDX11 was upregulated in HCC and promoted cell proliferation and migration *via* PI3K/AKT pathway (17), but failed to identify the direct downstream effector of DDX11. In our study, DDX11 interacted with EZH2 to enhance its protein stability by avoiding the ubiquitination-mediated protein degradation. As a result, DDX11 was able to suppress the expression of p21 in a p53-independent manner to facilitate the cell cycle progression. Taken together, these findings suggest DDX family proteins could be used as potential prognostic factors, and play important roles in the regulation of HCC progression.

Our study further identified E2F1 as one of the upstream regulators of DDX11, which was in line with the previous study (17). As a transcriptional factor, E2F1 participated in the tumor growth and metastasis in many types of human cancers, including HCC (26). Interestingly, EZH2, the downstream of DDX11 in our study, was demonstrated as one of the direct transcriptional targets of E2F1. EZH2 cooperates with E2F1 to enhance its transcriptional activity and control the mRNA expression of genes involved in the regulation of tumor aggressiveness *via* direct protein binding (27–29). Our *in vitro* experiments showed that EZH2 could increase the mRNA expression of DDX11, which was significantly attenuated by the knockdown of E2F1. Thus, E2F1 and EZH2 formed a positive feedback loop to upregulate the expression of DDX11 in HCC cells. Other studies revealed that DDX11 expression was modulated by other factors, including long noncoding RNA LncRNA DDX11 antisense RNA 1 (DDX11-AS1) (15). The critical role of DDX11-AS1 in HCC and colorectal cancer has been reported (30, 31). In osteosarcoma cells, DDX11-AS1 upregulated the expression of DDX11 *via* sponging miR-873-5p (15). It should be noted that DDX11-AS1 was also able to interact with EZH2 to repress the expression of LATS2 in HCC (30). However, the relationship of DDX11-AS1, DDX11 and EZH2 requires further investigation to disclose. Collectively, our data suggest DDX11 as a promising prognostic factor and oncogene in HCC *via* a E2F1/DDX11/EZH2 axis.

## Data Availability Statement

The raw data supporting the conclusions of this article will be made available by the authors, without undue reservation.

## Ethics Statement

The studies involving human participants were reviewed and approved by the Institute Research Ethics Committee of The Affiliated Hexian Memorial Hospital of Southern Medical University. The patients/participants provided their written informed consent to participate in this study.

## Author Contributions

Conception and design of the study: CZZ, HS. Generation, collection, assembly, analysis of data: SGS, QLL, MFZ, PWZ. Drafting and revision of the manuscript: SGS, CZZ, HS. Approval of the final version of the manuscript: all authors. All authors contributed to the article and approved the submitted version.

## Funding

This study is supported by National Natural Science Foundation of China (No. 81872266 and 81872387) and Medical Health Science and Technology Project of Panyu District of Guangzhou (No. 2019-Z04-04).

## Conflict of Interest

The authors declare that the research was conducted in the absence of any commercial or financial relationships that could be construed as a potential conflict of interest.
