# Supplementary material for: An E2F1/DDX11/EZH2 Positive Feedback Loop Promotes Cell Proliferation in Hepatocellular Carcinoma
Source: Front Oncol. 2021 Feb 5;10:593293. doi: 10.3389/fonc.2020.593293 (PMC7892623; doi:10.3389/fonc.2020.593293)
Supplement: Supplementary file 1 [file DataSheet_1.pdf]

Supplementary data

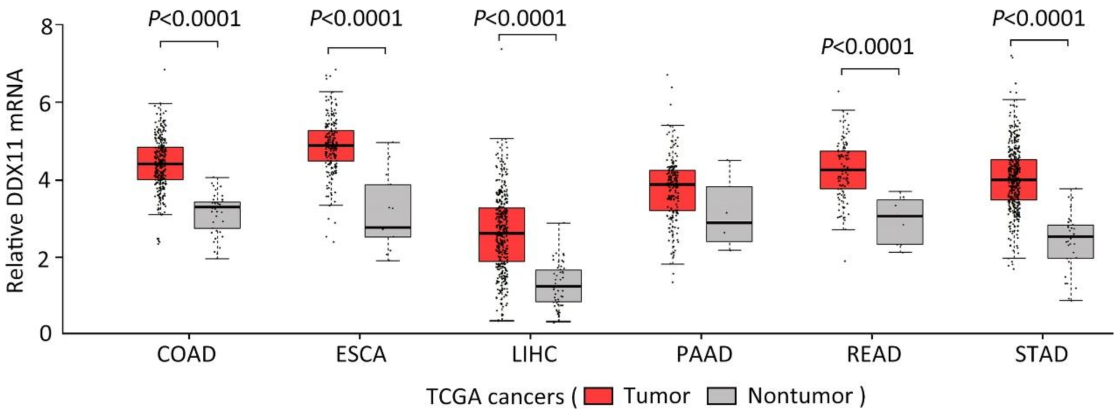

Supplementary figure 1. The expression of DDX11 mRNA in gastrointestinal cancers in TCGA. COAD, Colon adenocarcinoma; ESCA, Esophageal carcinoma; LIHC, Liver hepatocellular carcinoma; PAAD, Pancreatic adenocarcinoma; READ, Rectum adenocarcinoma; STAD, Stomach adenocarcinoma.

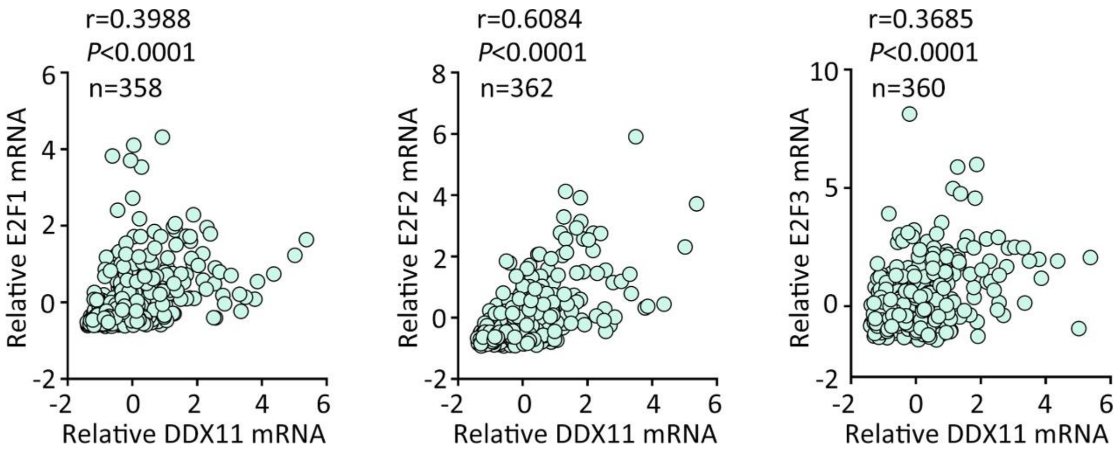

Supplementary figure 2. The correlation of E2F transcriptional factors and DDX11 in TCGA samples.

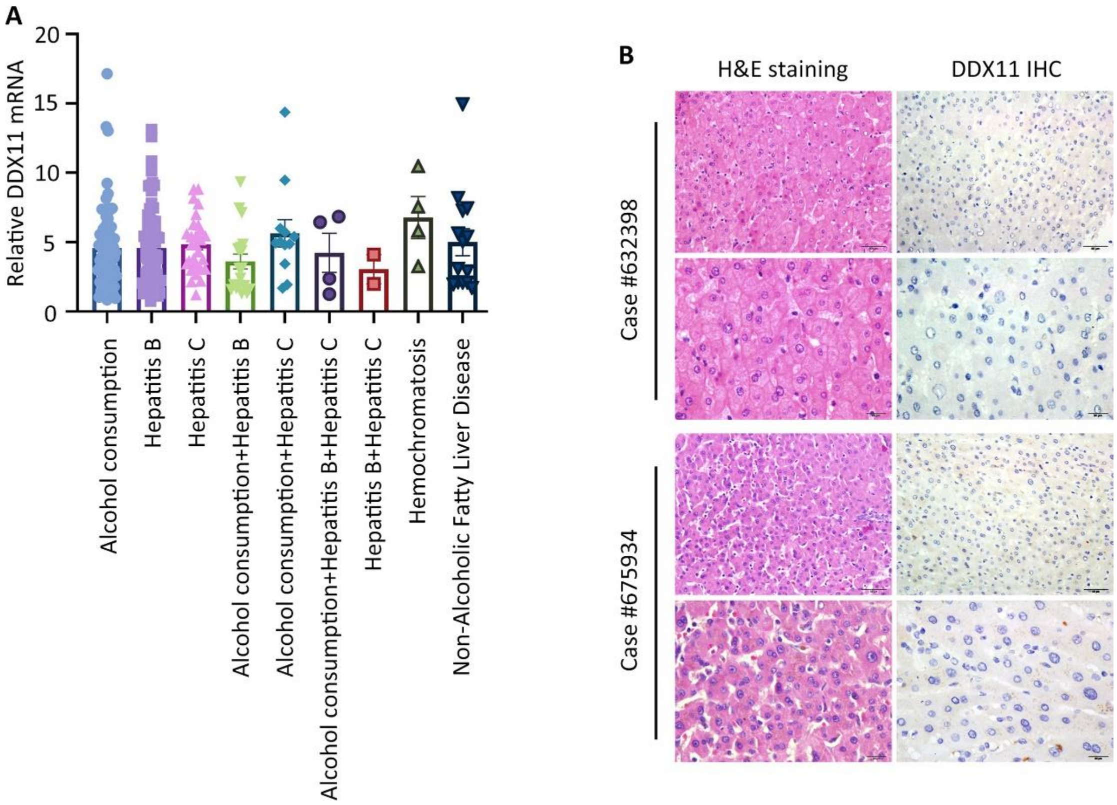

Supplementary figure 3. A. Association of DDX11 expression and HCC risk factors in TCGA patients with HCC. B. DDX11 protein expression in cirrhotic tissues adjacent to HCC in SYSUCC cohort.
